# Protein phosphatases in chromatin structure and function^☆^

**DOI:** 10.1016/j.bbamcr.2018.07.016

**Published:** 2019-01

**Authors:** Raquel Sales Gil, Paola Vagnarelli

**Affiliations:** Colleges of Health and Life Science, Research Institute for Environment Health and Society, Brunel University London, London UB8 3PH, UK

**Keywords:** Phosphatases, Chromatin remodelling, Histone phosphorylation, Epigenetics, Transcription regulation

## Abstract

Chromatin structure and dynamics are highly controlled and regulated processes that play an essential role in many aspects of cell biology. The chromatin transition stages and the factors that control this process are regulated by post-translation modifications, including phosphorylation. While the role of protein kinases in chromatin dynamics has been quite well studied, the nature and regulation of the counteracting phosphatases represent an emerging field but are still at their infancy. In this review we summarize the current literature on phosphatases involved in the regulation of chromatin structure and dynamics, with emphases on the major knowledge gaps that should require attention and more investigation.

In eukaryotic cells, DNA is wrapped around proteins called histones to form a structure termed the nucleosomes, the basic unit of chromatin. The central histone octamer consist of two H2A/H2B heterodimers and two H3/H4 heterodimers and it is connected to the adjacent nucleosome core through a linker DNA, usually associated with the linker histone protein H1. Nucleosomes are organized into 10 nm chromatin fibres that adopt various secondary structures that are amenable to interdigitated packing, including a loose zig-zag. This leads to a hierarchy of globular chromatin domains that allow for effective functional compartmentalization of the genome (topologically associating domains (TADs) [1] and A- and B-compartments) [2] that simultaneously compact the long 10-nm fiber to fit in the nucleus [3].

At the beginning of mitosis, Condensin II starts the shortening of the mitotic chromosomes by generating 200–400 kb loops that are then subdivided into smaller 80 kb loops by Condensin I; the loops then acquire a helical arrangement [4]. This organization needs to be dismantled at the end of division when chromatin de-condenses and TADs are correctly re-established to allow the subsequent cell cycle to begin.

The highly dynamic chromatin structure during the cell cycle is essential for many processes that safeguard genome stability such as DNA replication and repair, gene expression and chromatin segregation. Many factors are involved in controlling chromatin organization, ranging from histone modifications to ATP-dependent chromatin remodelling complexes and non-histone chromosome proteins. The majority of these factors are susceptible to reversible post-translational modifications (PTM), including phosphorylation, that regulate their functions. In this review, we will focus on the role of protein phosphatases in the regulation of chromatin organization and dynamics involved in several aspects of chromatin biology, namely chromatin structure (summarised in Table 1), DNA replication and repair, epigenetics, and gene expression.

**Table 1:** Summary of the known kinases and phosphatases involved in chromosome structure regulation.

	Kinases	Function	Phosphatases	Function
Chromatin condensation	CDK1	Anchoring site for PLK1	PP2A	Condensin II binding to chromatin
	PLK1	Condensin II binding to chromatin	PNUTS/PP1	Chromosome de-condensation
	Aurora B	Condensin I binding to chromatin	Repo-man/PP1	Chromosome de-condensation
Telomeres	ATM (Tel1)	Recruitment of telomerase to telomeres	Rif1/PP1	Inhibits Tel1 recruitment
	DNA-PK	Telomeres protection	PP2A	Inhibits telomerase recruitment to telomeres
Chromosome periphery	CDK1	Phosphorylation of perichromosomal proteins	Ki-67/PP1	Required for chromosome periphery formation
	CK-II			
	PLK1			
Centromeres/kinetochore	CDK1	Block CENP-A deposition to centromeres	PP1	Enhance CENP-A deposition on centromeres
	Aurora A			
	Aurora B			

## 1 Phosphatases in the regulation of chromosome structure

### 1.1 Chromatin condensation/de-condensation in mitosis

When cells enter mitosis, chromatin condenses and re-models into distinct rod-shaped structures known as mitotic chromosomes. The condensin complexes are key to the correct organization of mitotic chromosomes (reviewed in [5]). In vertebrates two condensin complexes contribute to the chromosome condensation pathway: condensin I and condensin II [4,6]. They share the same two core proteins (SMC2 and SMC4), but differ in the other three components of the complex: CAP-H, CAP-G and CAP-D2 for condensin I, and CAP-H2, CAP-G2 and CAP-D3 for condensin II. Condensin II is nuclear and can bind to chromosomes in early prophase, whereas condensin I is cytoplasmic and cannot bind chromosomes until nuclear envelope breaks down. The phosphorylation status of condensin is critical for its activity [7]; while protein kinases involved in mitotic entry phosphorylation and in the activation of condensin I and II have been extensively studied, the counteracting phosphatases remain elusive. At the beginning of mitosis CDK1 (Cyclin-Dependent Kinase 1) phosphorylates Thr1415 of the CAP-D3 subunit of condensin II, which serves as an anchoring site for PLK1 (Polo-like kinase 1), which in turn phosphorylates the condensin II complex at Ser288 and enables its binding to chromatin [8,9]. Aurora B, on the other hand, is the kinase responsible for phosphorylating condensin I and stimulating its binding to chromosomes after nuclear envelope breaks down, stabilizing the condensed status of chromosomes [10,11].

PP2A (Protein Phosphatase 2A) also plays a role in condensin II binding to chromosomes; in fact, addition of Okadaic Acid (OA) (an inhibitor of Protein Phosphatase 1 (PP1) and PP2A) to chromosomes assembled using mitotic *Xenopus* egg extract, resulted in the loss of condensin II from the chromosomes but it did not affect the chromosomal association of condensin I or topoisomerase II. Addition of two purified PP2A complexes back to the PP2A-depleted *Xenopus* eggs (the PP2A core dimer (PP2A-Aα and PP2A-Cα) and the B56γ trimer (core dimer and PP2AB′/PR61/B56γ)) was able to rescue the chromosomal association of condensin II, however no rescue was obtained with the B55α trimer (core dimer and PP2A-B/PR55/B55α). Interestingly, both the PP2A dimer containing PP2A^*wt*^ and the one with the PP2A^*CD88N*^ mutant (which has a weak phosphatase activity) were able to rescue the localisation of condensin II suggesting that the chromosomal association of PP2A, but not its phosphatase activity, is essential for the chromosomal targeting of condensin II [12]. Depletion of PP2A by siRNA in HeLa cells also disrupted the binding to chromatin of condensin II, but not of condensin I, indicating that this mechanism is conserved from *Xenopus* to humans. In addition, PP2A depletion also showed impairment of KIF4-chromosome binding, but not of Aurora B. KIF4 is a chromokinesin involved in mitotic chromosome assembly [13], reinforcing the important role of PP2A on chromosome structure [12].

Condensin activity is inhibited by dephosphorylation of three of its subunits, including CAP-G, by PP2A. XCAP-D2, XCAP-G, and XCAP-H, three subunits of condensin in *Xenopus*, have been shown to be hyperphosphorylated in mitosis and these phosphorylations regulate the complex affinity for chromatin [8,14].

PP2A interacts with TATA binding protein (TBP) in extracts of mitotic cells. TBP exists in cells as part of the TFIID complex and remains bound to DNA during mitosis; at these mitotic sites it recruits PP2A. Since TBP interacts with condensin as well, it promotes its dephosphorylation mediated by PP2A interaction and leads to the inactivation of condensin near these promoters to inhibit their compaction. This mechanism is part of gene bookmarking and therefore links PP2A to the preservation of the memory of gene activity through mitosis to daughter cells [15].

Due to the different kinases and multiple phosphorylated condensin sites during mitosis, it is difficult to believe that just one phosphatase is responsible for reversing the process, thus further research on condensin dephosphorylation and the role of phosphatases in chromatin condensation would be an interesting avenue to pursue.

Although the role of condensin complexes on chromosome assembly and segregation can't be argued, there must be other factors that are involved in chromosome condensation at mitotic entry, as condensin knockdown in chicken cells showed that chromosomes can still condense in the absence of condensin [[16], [17], [18]]. This factor, called RCA (regulator of chromosome architecture), enables condensation of chromosomes even in the absence of condensin until anaphase, where chromatin of condensin-depleted cells abruptly changes its structure resulting in abnormal chromosome segregation and cell death [17]. Repo-man/PP1 phosphatase complex was shown to be involved in regulating RCA activity, as normal chromosome segregation in condensin-depleted cells could be rescued by a Repo-man RAXA mutant, which is not able to bind and target PP1 to chromatin. CDK1 phosphorylates Repo-man at mitotic entry, which disrupts Repo-man/PP1 interaction and chromatin binding. At anaphase, CDK1 levels drop and dephosphorylated Repo-man is able to recruit PP1 to chromatin where it dephosphorylates RCA and triggers chromatin de-condensation [17,19].

Another example of the involvement of PP1 in chromosome condensation/decondensation comes from studies where the PP1 targeting subunit PNUTS (PP1 nuclear targeting subunit) was shown to accelerate chromosome decondensation in a PP1-dependent manner [20]. However, the nature of the substrates and the critical networks regulated by this complex are still unknown.

Chromosome condensation can be induced also outside the M phase using OA and calcyculin A, two potent PP1 and PP2A inhibitors. This state of chromatin is called Premature Chromosome Condensation (PCC). This phenomenon has gained considerable attention in recent years since it has been linked to genomic rearrangements and diseases [21,22]. Studies on the structure of PCC-induced chromosomes have shown that Condensin I and II individually are dispensable for PCC but the lack of both leads to condensation defects even outside mitosis [23]. Altogether these studies clearly demonstrate that active phosphatases are needed in interphase to actively maintain a de-condensed chromatin. However, the molecular details are still not known.

### 1.2 Regulation of telomeres

Telomeres are repeated DNA sequences situated at the chromosome ends that protect the DNA from deterioration and ensure genome stability. They mostly comprise of 6 to 8 base pairs stretches and the consensus sequence for vertebrates is TTAGGG. In each cell cycle, due to the end-replication problem, the telomeres get shorter and the enzyme telomerase needs to be recruited to the chromosomes to elongate and extend the TG-rich terminal repeat sequences. Studies in yeasts have shown that the action of Tel1 kinase (ATM, ataxia-telangiectasia mutated, in mammals) is crucial for the recruitment of telomerase to short telomeres and thus it is to be expected that this process is counteracted by protein phosphatases. In fact this is the case; PP1, through interaction with the targeting subunit Rif1, is essential for the suppression of TG repeat elongation via Tel1 recruitment inhibition, and this function is independent of DNA replication. Defective Rif1/PP1 interaction leads to a long-telomere phenotype although it does not compromise the binding of Rif1 to telomeres, highlighting the crucial role of the phosphatase activity [24]. PP2A was also shown to be involved in regulating the recruitment of telomerase to telomeres. In this case, PP2A dephosphorylates the telomere binding protein Cdc13 in yeasts and inhibits telomerase recruitment at the G2/M transition, ending telomere replication and enabling progression through mitosis [25]. The PP2A scaffolding subunit PR65α is also involved in the regulation of telomere maintenance as it interacts with the catalytic subunit of telomerase, hTERT, and inhibits its activity in vivo and in vitro by regulating hTERT subcellular localization [26].

### 1.3 Chromosome periphery

Mitotic chromosomes are surrounded by a complex network of proteins and RNA molecules, mainly derived from the nucleolus, called the chromosome periphery. Although the function and characteristics of this layer still remain unclear, it is now known that Ki-67, a PP1-interacting subunit, is required for its formation (see [27,28] for review); Ki-67 depleted cells fail to associate some of the main components of the chromosome periphery, including NIFK, B23, and nucleolin [29]. Loss of the perichromosomal layer did not show any effect on the mitotic chromosome structure but produced chromosomes collapsing into a single chromatin mass after nuclear envelope disassembly and prevented chromosome motility and efficient interactions with the mitotic spindle. The function of Ki-67 in this respect is to act as a biological surfactant to disperse mitotic chromosomes by forming an electrostatic charge barrier [30]. However some of these functions seem to be independent from its association with PP1 [29] thus opening the question if any other PP1-associated functions are to be discovered.

Furthermore, the nuclei formed following Ki-67 depletion are smaller than the control and they possess a single large nucleolus, indicating a possible role of the chromosomal periphery also in the organization of the nucleolus [29]. The mechanism by which Ki-67 binds to chromatin and regulates the formation of this compartment is still under investigation, although Ki-67 association with the chromosomal periphery during mitosis seems to be regulated by the p150 subunit of CAF-1 (Chromatin Assembly Factor 1), as shown by shRNA experiments in HeLa cells [31]. Altogether indicate that Ki-67 is an important player on chromosome periphery formation that could potentially be used to unravel the functional characteristics of this mysterious chromosomal layer.

### 1.4 DNA replication

As the cell divides, the genome has to be replicated and passed onto the new generation of cells to ensure that the genetic information is conserved over generations. During DNA replication, the existing chromatin structure is disrupted by the passage of the replication fork to enable DNA polymerase access to the template DNA. At the end of G1, a pre-replication complex (pre-RC) is formed, composed of 6 ORC proteins, Cdc6, Cdt1 and Mcm2–7, which is activated at the beginning of the S phase by several protein kinases, including CDKs and DDK (Dbf4-dependent kinase). The role of protein kinases on DNA replication has been extensively studied by several groups (reviewed in [32]), although only a few phosphatases have been discovered with a function in this process. Data from different labs using *Xenopus* egg extracts and *Saccharomyces cerevisiae* showed that PP2A is important for the G1/S transition and the initiation of DNA replication, but not for elongation, as it is essential for the loading of Cdc45 to the pre-RC, which then forms the initiation complex [[33], [34], [35]]. Data on human cells also showed the importance of PP2A inhibition on replication origin firing, as failure to inhibit PP2A during S-phase leads to increased degradation of Treslin, a crucial factor for pre-RC assembly and Mcm helicase activation, and results in an extended S-phase mediated by a decrease on the number of fired replication origins [36].

A role for PP1 on DNA replication has also been reported. Through its regulatory subunit Rif1, PP1 stabilizes the ORC (origin recognition complex) at G1 and has a positive role in supporting origin licencing. However, at the S phase, PP1/Rif-1 complex acts as repressor of origin activation by counteracting DDK and inhibiting phosphorylation of Mcm4. While the latter has been reported in several organisms including yeast and humans [[37], [38], [39]], the role of PP1 in protecting ORC1 degradation has only been reported in human cells [39]. In this way, PP1 acts as a regulatory protein to limit the number of origins fired. Studying the other phosphatases involved and the specific targeted sites will allow us to have new insights in this important process of the cell cycle and ways to address any DNA replication problems that might arise.

DNA polymerase is the enzyme responsible for DNA replication. There are many different types of DNA polymerases and it has been reported that most of them need to be phosphorylated at specific sites to initiate replication [[40], [41], [42]]. One of these studies showed that DNA polymerase δ is phosphorylated by CK2 in three of its four subunits: p120, p68, and p12. It was also shown that these phosphorylations are substrates for PP1 [43]. Although no other phosphatase apart from PP1 has been identified in regulating DNA polymerases, it is to be expected that, based on the level of phosphorylation of this enzyme, it cannot be regulated by the activity of just one phosphatase.

After DNA is replicated, chromatin structure needs to be re-established. Two pathways have been seen to contribute to this re-establishment: generation of parental histones that can be recycled behind the fork, and de novo deposition of newly synthetized histones. In relation to this, it has been shown that H3K27me3 recruits PRC2 to sites of DNA replication maintaining H3K27me2/3 and ensuring that the heterochromatin mark is replicated and deposited on the newly formed DNA. The phosphorylation status of the nearby H3S27 is important for PRC2 binding to chromatin [44]; as Repo-man/PP1 has been shown to target H3S27 in mitosis [45], it could be that this complex also plays a role in maintaining heterochromatin environment after DNA replication. Similarly, Repo-man/PP1 might act by keeping H3S10 dephosphorylated, so HP1 (Heterochromatin Protein 1) can interact with H3K9me2/3 during replication and recruit the methyltransferase Suv39. Suv39 methylates H3K9 in adjacent nucleosomes containing unmodified H3, maintaining the heterochromatin environment after replication [46]. However, as far as we are concerned, the role of PP1 in maintaining chromatin structure after DNA replication has not been studied.

### 1.5 DNA repair

DNA integrity and stability are constantly challenged by environmental agents and stressors that may cause different grades of DNA damage. The cell has mechanisms and DNA damage checkpoints essential to recover from lesions and maintain genome integrity. Four DNA damage checkpoints exist through the cell cycle and they all need to be inactivated after DNA repair to ensure cell cycle progression. DNA repair has been extensively studied in *Saccharomyces cerevisiae* and, while there are plenty of studies about how the checkpoints are activated, there is not much known about how they are inactivated. The DNA damage response (DDR) starts with the activation of the ATM and ATR (ATM and Rad3-related) kinases, which phosphorylate a number of proteins that will activate the DDR cascade. ATM and ATR activation are regulated by the action of several phosphatases, including PP2A, PP1, Wip1 (Wild-type p53-Induced Phosphatase 1), and PP5. PP2A has been demonstrated to interact with ATM, and inhibition of the phosphatase induced auto-phosphorylation of ATM at Ser1981, although it did not correlate with an increase of the kinase activity, suggesting that PP2A is not dispensable for ATM activation. However, ionizing radiation was able to disrupt PP2A-ATM binding and this dissociation required ATM kinase activity, suggesting that ATM auto-phosphorylation or ATM-mediated phosphorylation of PP2A could be important for complex dissociation. After IR exposure, PP2A would dissociate from ATM enabling its auto-phosphorylation and interaction with targeted protein to start DNA damage repair [47]. Similarly, PP5 also interacts with ATM in a DNA damage-dependent manner and regulates its activation, as down-regulation of PP5 attenuated DNA damage-induced ATM activation [48]. Furthermore, Wip1 was able to dephosphorylate ATM at Ser1981 and decrease its activity [49]. These phosphatases are also important for the regulation of other DDR-related factors, including Chk1, Chk2, p53, BRCA1, and pRB [50].

### 1.6 Centromere/kinetochore regulation

Chromosome segregation is facilitated by the formation of kinetochores at the centromeric regions of the chromosomes that enable microtubule attachment to pull sister chromatids apart. These proteinaceous structures are also involved in the surveillance mechanism known as the spindle assembly checkpoint (SAC); this mechanism controls that sister chromatids are properly attached to the mitotic spindle and signals when it is safe for the cell to progress to anaphase. Once more, the right balance between kinases and phosphatases is critical for the formation of kinetochores and the proper activation of SAC.

Since the role of PP1 and PP2A in the SAC has been extensively studied and excellent reviews are available on the topic [51,52], we would like to highlight here only the aspect related to the organization and structure of the centromeric chromatin.

**Fig. 1:**
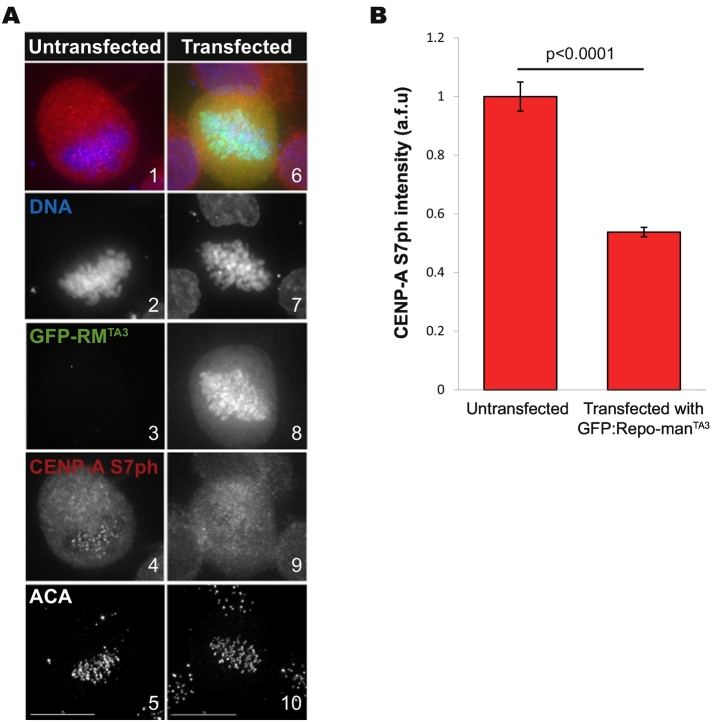
Overexpression of GFP:Repo-man^TA3^ (GFP-RM^TA3^), a hyperactive form of Repo-man, causes dephosphorylation of CENP-A Ser7ph in early mitosis (compare panels 4 with 9). DT40 cells were transfected with GFP-RM^TA3^ then fixed and stained for CENP-A phSer7 (A). The intensity of CENP-A phSer7 at each centromere was measured and quantified (B). Scale bars: 15 μm. Experiment conducted by Thomas William Monteiro Crozier.

At the core of the centromere is CENP-A, a centromere specific H3 variant that replaces histone H3 only at the centromeric domain. CENP-A deposition does not occur during S phase but only after cells have divided, in G1. CDK1 inactivation and phosphatase activity are essential for its incorporation [53]. At late G2/early mitosis, Aurora A + Aurora B and CDK1 phosphorylate CENP-A at Ser7 and Ser68, respectively [54,55]. The function of Ser7 phosphorylation (Ser7ph) has been controversial but the general consent is that it is important for Aurora B localization at centromeres. In fact CENP-A mutants lacking this phosphorylation cannot rescue the mitotic defects caused by the loss of endogenous CENP-A [56]. Ser7ph is reversed during mitotic exit and, considering that this residue is homologue to the Ser10 on the canonical H3, we hypothesised that Repo-man/PP1 was the dedicated phosphatase. In fact, this seems to be the case (Fig. 1); cells transfected with a hyperactive form of the Repo-man (GFP:Repo-man^TA3^) [63], do not present CENP-A ser7ph on mitotic cells. However the consequences of the lack of CENP-A de-phosphorylation are still under investigation.

Ser68 phosphorylation has been shown to impair CENP-A binding to HJURP, an assembly factor that mediates CENP-A deposition to centromeres. During anaphase, PP1α antagonises this effect and removes the phosphate group to enable HJURP binding and proper loading of CENP-A to the centromeres [57]. Other phosphorylable sites of CENP-A have been described [54,58], although the responsible phosphatases that counteract this action have not been identified and would be an interesting field to pursue.

Within the SAC response, Aurora B destabilizes erroneous attachments and activates the SAC, which monitors the lack of tension. Shugoshin (in collaboration with histone H3Thr3 phosphorylation by Haspin kinase) is also important for the loading of Aurora B complex [59,60] to centromeres and ensures the bipolar attachment of kinetochores. The conserved SAC protein kinase Bub1 plays a role in chromosome congression and an auxiliary role in SAC activation by phosphorylation of H2AS121. This phosphorylation allows the binding of Shugoshin [61] and causes a reduction in pericentric chromatin dynamics [62] thus modulating the SAC response. The counteracting phosphatases for H3Th3ph is Repo-man/PP1 [63,64] but there is no candidate for the H2AS121ph as yet.

## 2 Protein phosphatases in the regulation of gene expression

### 2.1 RNA polymerase II

RNA polymerase II (RNAPII) possesses an evolutionary conserved C-terminal domain (CTD) made up of multiple 7 amino acid repeats that is susceptible to phosphorylation. CTD phosphorylation status, highly dynamic during the transcription cycle, is critical for many steps, as it acts as a scaffold for diverse nuclear factors. The serines mentioned in the text are numbered according to their position within the CTD repeat. Phosphorylation of Ser5 is important to recruit enzymes that cap the 5′ end of the transcript, whereas phosphorylation of Ser2 activates elongation and splicing. Subsequent dephosphorylation of these sites is needed to finish transcription and enable detachment of RNAPII of the gene. Fcp1 phosphatase specifically dephosphorylates Ser2 [65], while Ssu72 and Rtr1 in yeast, and SCP1 and RPAP2 in mammals are responsible for Ser5 dephosphorylation (see [66] for review). PNUTs/PP1 was also shown to target Ser5ph, as PNUTs/PP1 co-localize with RNAPII at transcriptionally active sites and *Drosophila* PNUTs mutants increased the levels of RNAPII Ser5ph levels but not of Ser2ph [67]. Ser7 on RNAPII is also susceptible to phosphorylation, although the specific role of this modification is not understood. Mutation of Ser7 to glutamate, a phosphor-mimic, is lethal thus indicating that Ser7 dephosphorylation is crucial for cell viability; Ssu72 is the phosphatase responsible for this dephosphorylation [68]. The transcription factor Sp3 inhibits the transition of paused RNA Pol II to productive elongation at the promoter of the cyclin-dependent kinase inhibitor p21CIP1 and other Sp3-repressed genes by recruiting PP1 to the promoter [69]. For an extended review on RNAPII dephosphorylation please see [70].

PP1 is also implicated in other aspects of transcription and in fact all the three PP1 isoforms (PP1α, PP1β, PP1γ) have been found bound to promoters in HeLa cells by methylation-based DamID profiling. It seems that this binding is not direct but it is regulated by other PP1 interacting subunits (PIPs); PNUTS and NIPP1 (Nuclear inhibitor of PP1) show the highest degree of overlaps with PP1 at promoters but the majority of PP1 binding sites do not overlap with any of the major chromatin-associated subunits, suggesting that PP1 is recruited to promoters by other yet uncharacterized targeting subunits [71]. In addition, in vivo experiments in *Drosophila* showed that PNUTS/PP1 is important for development and loss of PNUTs affects the transcription of the majority of genes in developing larvae [67]. NIPP1 has been shown to affect the expression of proliferation-related genes by controlling the phosphorylation status of EZH2 [72,73].

### 2.2 Protein phosphatases in epigenetic regulation

**Fig. 2:**
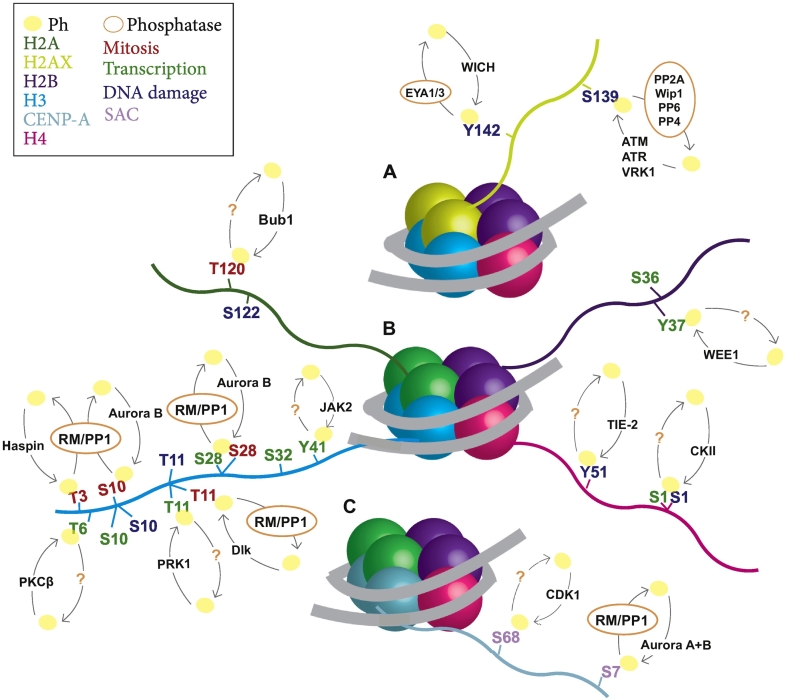
Histone phosphorylation regulation model. The image shows the main phosphorylated residues of histones during different stages of the cell cycle, with the responsible kinases and phosphatases involved. Residues in red indicate phosphorylation during mitosis; in green during DNA transcription; in blue during DNA damage; and in light purple during Spindle Assembly Checkpoint (SAC). Histone H2AX is coloured in light green (A), H2A in dark green (B), H2B in purple, H3 in blue, H4 in pink, and CENP-A in light blue (C). Phosphatases involved are highlighted in orange. RM: Repo-man: PP1, PP2A, PP5, PP6: Protein phosphatase 1, 2A, 5, 6, respectively. ATM: ataxia-telangiectasia mutated; ATR: ataxia telangiectasia and Rad3-related protein; VRK: Vaccinia-related protein kinases; JAK: Janus kinase; PKC: Protein kinase C; Dlk: death-associated protein (DAP)-like kinase; PRK: Phosphoribulokinase: CKII; Casein Kinase II.

Chromatin remodelling and function is also controlled by changes in DNA methylation, histone variants and their post-translational modifications. These mitotically transmissible but potentially reversible modifications are referred to as epigenetics. The epigenetic landscape is crucial for chromatin structure and dynamics, and many epigenetic marks have been described for different stages of the cell cycle. Histones are modified at several sites and phosphorylations are among those modifications (Fig. 2). Although much attention has been placed on the enzymes that reverse the acetylation, methylation, ubiquitination (generally referred to as “epigenetic erasers”) much less emphasis has been given to the identification of the enzymes that erase the phosphorylation marks. Only recently new studies have shed light on how some of these phosphorylations are removed but it is still quite an uncharted territory.

Here we summarize the specific histone phosphorylation modifications that are regulated during the cell cycle (see also [74]), emphasizing the role of protein phosphatases in the regulation of chromatin remodelling (Fig. 2).

#### 2.2.1 Histone phosphorylation during mitosis

As cells enter mitosis, Aurora B kinase is activated and phosphorylates Ser10 and Ser28 on histone H3. This phosphorylation starts at pericentromeric heterochromatin and it spreads along the entire length of chromosomal arms [75]. H3S10 and H3S28 phosphorylation starts in early prophase coinciding with chromosome condensation, reaches its maximum in metaphase, and the levels decrease as cells enter anaphase. Phosphorylation of H3T3 by Haspin is also important in mitosis, especially for kinetochore-microtubule attachment and error correction, as it serves as a docking site for Survivin, which mediates CPC (Chromosomal Passenger Complex) accumulation at the centromeres and proper Aurora B activation. Bub1 kinase was also shown to cooperate with Haspin in targeting CPC to the centromeres by phosphorylation of H2AT120, which appears to regulate H3T3ph distribution [76]. All these mitotic phosphosites of Histone H3 (T3, S10 and S28) are removed during mitotic exit by the Repo-man/PP1 complex. At mitotic entry, the activation of CDK1 phosphorylates Repo-man at different sites and decreases its affinity to PP1 and chromatin [63,77,78]. In early anaphase, when CDK1 is inactivated, PP2A and PP1 contribute to Repo-man de-phosphorylation, enabling its binding to chromatin and recruitment of PP1 to chromosomes, where it dephosphorylates H3T3, H3S10, and H3S28 [45,63,77]. H3T3 dephosphorylation results in CPC delocalization from chromatin to the central spindle to allow proper chromosome segregation. Dephosphorylations of H3S10 and H3S28 play a role on chromatin transcription regulation by controlling the chromatin binding of HP1 and PRC2, respectively (seen in more detail later in this review). [45,63,64,77]. Although the phosphorylation of S10 and S28 do correlate with chromosome condensation, they are not required for it in vertebrates [79]. However, mutation of Ser10 to a non-phosphorylable residue in *S*. *pombe* presents several mitotic defects, including impaired chromosome segregation [80], but the same mutation in *S*. *cerevisiae* does not show any mitotic impairment [81], indicating that the importance of this histone modification might vary between organisms.

Furthermore, H3T11 is also phosphorylated in mitosis by the death-associated protein (DAP)-like kinase (Dlk) and localises to the centromeres from prophase to early anaphase; this modification appears to have a role in kinetochore assembly [82].

Histone H1 is also regulated in a cell cycle-dependent manner, and its phosphorylation significantly increases during mitosis and S phase. Several phosphorylated sites have been identified for H1 and they have been related with chromatin decondensation rather than chromatin condensation [83]. H1 was shown to bind HP1 and stabilize the compacted chromatin structure, and its phosphorylation by CDK2 disrupts H1-HP1 binding, destabilizing chromatin and enabling proper cell cycle-progression [84]. It is believed that the number of phosphorylated sites in H1 is the important aspect for the functions rather than the specific residues themselves [85]. However, the phosphatases acting on the H1 sites are not known.

#### 2.2.2 Histone phosphorylation in DNA damage response

An important epigenetic mark for DNA damage response (DDR) is the phosphorylation of the histone variant H2AX at Ser139 by ATM, ATR, and VRK1 (Vaccinia related kinase 1) [[86], [87], [88]]. H2AXS139ph, commonly referred to as γH2AX, is involved in diverse DDR pathways, including non-homologous end joining (NHEJ), homologous recombination (HR), and replication-coupled DNA repair, and it is widely used as a marker for double strand breaks DNA-damage. After 30 min of DNA damage, ATM/ATR are recruited to the chromatin, where they phosphorylate H2AX at Ser139, and the mark then spreads around the chromatin to decondense chromatin and serves as a signalling platform to recruit specific DNA damage factors [74,89]. VRK1 activation upon IR is also required for the formation of γH2AX foci, which could be explained by the fact that VRK1 knockdown resulted in lack of ATM activation [88]. Once DNA damage has been repaired, γH2AX needs to be removed from the chromatin to ensure proper progression through the cell cycle. Some controversies exist in whether γH2AX is replaced by other histones, although there is evidence that γH2AX is dephosphorylated after DNA is repaired. In yeast, γH2AX is dephosphorylated by the HTP-C phosphatase complex (histone H2A phosphatase complex), enabling DNA damage checkpoint recovery [90]. In mammals, many phosphatases seem to be involved in γH2AX dephosphorylation, including PP2A, Wip1, PP6, and PP4, as shown by siRNA experiments where inhibition of any of the phosphatases resulted in increased γH2AX foci persistence, inefficient DNA repair, and hypersensitivity to DNA damage [[91], [92], [93], [94], [95]]. This is in agreement with the fact that all these phosphatases are involved in ATM activity regulation, as we have previously seen in this review. In the case of PP6, DNA-dependent protein kinase (DNA-PK) is responsible for recruiting PP6 to damaged sequences to dephosphorylate γH2AX and release the cell from G2/M checkpoint [95], whereas in the case of Wip1, it seems that ATM and Chk1/2 kinases regulate Wip1 activity through the phosphorylation of p53. p53 phosphorylation indirectly increases Wip1 levels, which in turn dephosphorylates p53 itself, γH2AX, and some other checkpoint substrates leading to checkpoint inactivation. To date, the specific checkpoint substrates dephosphorylated by Wip1 are not known [92,93].

H2AS122 also seems to be phosphorylated upon DNA damage and, although it is not crucial, it facilitates survival in the presence of DNA damage [96].

H2AX can also be phosphorylated in Tyr142 (Y142), although it is found at very low levels in cells (<1%) [97]. Y142 is phosphorylated by the WICH complex and dephosphorylated by EYA1/3 upon DNA damage, contrary to γH2AX [98]. H2AXY142 dephosphorylation is important for γH2AX stability and failure to dephosphorylate this residue results in a decreased accumulation of DDR factors at DNA damage sites.

Ser10 and Thr11 on H3 are also susceptible to modifications upon DNA damage; in mouse, both residues are dephosphorylated after UV radiation-induced DNA damage and re-phosphorylated after repair [99,100]. However, the specific function of these alterations and the responsible phosphatases are yet to be discovered.

Phosphorylation of histone H4 at Ser1 by CKII also plays a role in DDR [101]. ChIP experiments showed that this phosphorylation is restricted to small regions around the DNA damage sites and appear later than γH2AX, indicating a role in later stages of the DDR. Mutations to a non-phosphorylable residue do not increase sensitivity to genotoxic agents, thus H4S1ph does not seem to be essential for DNA repair [102,101].

Another recently identified DNA damage-related histone modification is the phosphorylation of Tyr51 on H4 by TIE-2 upon ionizing radiation. H4Y51ph is recognized by ABL1, a kinase involved in DDR, and other DDR-related proteins, indicating a positive role of this modification in DNA repair machinery [103].

Identifying the phosphatases involved in DDR response, as well as the phosphatases that regulate chromatin structure during DNA repair will allow us to understand how DNA repair is controlled in space and time in the cell and maybe shed light on why, in some cases, DNA lesions escape the checkpoints and can pass into the next generation of cells, influencing genome stability and leading to different diseases.

#### 2.2.3 Histone phosphorylation associated with transcription regulation

Gene expression is highly regulated by chromatin organization and structure, and a large number of histone modifications have been linked to this specific process in response to several stimuli. H3S10, H3S28 and H2BS32 are phosphorylated upon specific growth factors stimulation and have been linked to the transcription of growth factor-responsive genes, including *c*-*fos*, *c*-*jun*, and *c*-*myc* [[104], [105], [106]]. Specifically, increased levels of H3S10ph were found in Ras overexpressing cells [104], and levels of H2BS32ph are high in EGF treated cells and low in serum-starved cells [105]. The pleckstrin homology domain leucine-rich repeat protein phosphatase (PHLPP) seems to counteract this activity by dephosphorylating several histone residues, including H3S10ph, H3S28ph, and H2AS139ph, suppressing growth factor signalling [107]. According to this, H3S10ph seems to be dephosphorylated by MKP-1 (MAP kinase phosphatase 1) upon VEGF and thrombin stimulation, which might act as a temporal repressor or regulator of transcriptional machinery to the promoters of inflammatory genes [108].

Phosphorylation of Thr11 and Thr6 on H3 upon androgen stimulation by PRK1 and PKCβ, respectively, is linked to regulation of androgen-associated genes by controlling H3 methylation levels. H3T11ph and H3T6ph promote removal of the repressive H3K9me mark by the Jumonji C (JmjC) domain-containing protein JMJD2C4 [109,110]. Furthermore, H3T6ph was seen to prevent LSD1-mediated removal of H3K4me1/2, a mark of actively transcribed chromatin [110]. Upon DNA damage, H3T11ph is dephosphorylated, leading to a decrease in transcription of genes related to cell-cycle progression [99].

Another histone modification that influences transcription is the phosphorylation of Tyr41 on H3 by Janus Kinase 2 (JAK2), which has been seen to disrupt HP1α binding to chromatin, leading to the transcriptional activation of JAK2-regulated genes [111].

Studies on position-effect variegation (PEV) in *Drosophila* identified PP1 as a suppressor of variegation, *Su*(*var*)*3*–*6*, which support the idea of PP1 as a heterochromatin regulator. Chromosomal rearrangements or transpositions can lead to euchromatic genes being moved to nearby heterochromatic regions, which might suppress the expression of genes that are usually activated. Diverse mutagenesis experiments identified several genes that enhanced the expression of variegated genes (enhancers of variegation, *E*(*var*)), and some that suppressed its expression (suppressors of variegation, *Su*(*var*)). *Su*(*var*)*3*–*6*, which encodes for PP1, was seen as one of these genes that, once mutated, suppressed the expression of variegated genes, meaning that in normal conditions *Su*(*var*)*3*–*6* acts as an enhancer of heterochromatin formation [112].

HP1 is a known repressive marker for gene transcription and heterochromatin formation, as it binds to histone H3 tri-methylated in lysine 9 (H3K9me3) and, by forming a bridge with another H3K9me3, condenses chromatin to form heterochromatin and represses gene expression [[113], [114], [115], [116]]. Other histone modifications have been linked with HP1 ability to bind chromatin. As commented above, H3S10ph prevents HP1 binding to chromatin, supporting the role of H3S10ph as a transcription activator. Repo-man/PP1 was shown to dephosphorylate H3S10ph at anaphase, allowing the HP1 re-binding to chromatin and maintaining a repressive chromatin status after mitosis. Repo-man depletion in human cells decreased levels of heterochromatin markers, namely H3K27me2/3, H3K9me3, and HP1, and increased the levels of H3K9ac, a marker for active chromatin, indicating an important role of Repo-man/PP1 on heterochromatin maintenance [45].

Polycomb groups (PcG) proteins are also a family of proteins that epigenetically alter chromatin in order to repress gene expression, mainly of genes related to proliferation and differentiation. There are functionally two different polycomb complexes: Polycomb Repressing Complex 1 and 2 (PRC1 and PRC2). One of the main components of PRC2 is EZH2 (Enhancer of Zeste 2), a methyltransferase responsible for di−/tri-methylation of histone H3 lysine 27 (H3K27me2/3). H3K27me2/3 is a marker for gene silencing important also for stabilizing HP1 binding to chromatin [117]. HP1 is also phosphorylated; the phosphorylations appear to mediate its DNA- or RNA-binding ability and could contribute to HP1 heterochromatin targeting. Moreover, studies using fly HP1α and fission yeast Swi6 demonstrated defective heterochromatic silencing in cells expressing a phosphorylation-deficient mutant HP1 [[118], [119], [120]]. No phosphatase has been identified that mediated the dephosphorylation of HP1.

As in HP1 chromatin binding, the nearby amino acid (H3S28) phosphorylation status is important for PRC2 binding to H3K27me2/3, and PP1 plays an important role in its regulation. In early mitosis H3S28ph is crucial for PRC2 interaction with DNA and cell proliferation. Two different phosphatases contribute to the maintenance of this epigenetic landscape. NIPP1, a PP1 regulatory subunit that inhibits PP1, can bind to phosphorylated EZH2 and block its dephosphorylation (normally carried out by PP1), thus keeping EZH2 bound to chromatin [72,73]. NIPP1 was also found to interact with another PRC2 component, EED (Embryonic Ectoderm Development), and to act as a transcriptional repressor of targeted genes [121,122]. At mitotic exit Repo-man/PP1 mediates the dephosphorylation of H3S28, allowing PRC2 binding to chromatin thus playing a role in gene expression regulation. Depletion of Repo-man in fact alleviates the transcriptional repression at several polycomb-repressed genes [45].

Other histone phosphorylations are linked to gene transcription regulation. ChIP experiments revealed a role of H2BS36ph on AMPK-mediated gene expression. Loss of H2BS36ph resulted in reduced expression and lower survival rates in response to metabolic stress. The nearby Tyr37 is also a target residue for phosphorylation by the WEE1 kinase, and it is important for the suppression of replication-dependent core histone gene transcription [123]. H4S1 is also phosphorylated upon transcription activation in a CKII-dependent manner and it regulates acetylation-dependent chromatin relaxation [102]. Additionally, H4S1 is a substrate of the sporulation specific kinase Sps1, and they are both enriched in the promoters of sporulation genes, identifying a new target for proper meiosis. A role of H1 overall phosphorylation has also been seen in chromatin relaxation to allow transcription probably by changing the charge of a small domain of H1, although the exact domain and the mechanisms are not clear [85].

Taken together it is apparent that histone phosphorylations are critically important for the regulation of gene expression and, although many studies have been conducted to elucidate the specific roles of the phosphorylation sites that regulate transcription, new research on this area should include the identification of the counteracting phosphatases responsible for removing the histone modifications to allow a clearer picture on how transcriptional homeostasis is maintained and regulated.

### 2.3 Phosphatases in transcription factor regulation

**Fig. 3:**
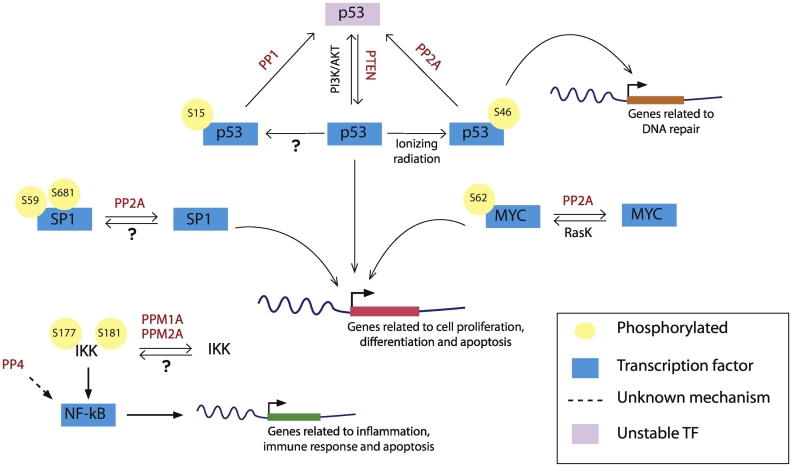
Phosphatases involved in transcription factor regulation. The graph shows the known phosphorylation sites of the main TF and the corresponded protein kinases (in black) and phosphatases (in red), indicating the type of genes that they regulate. See text for more details.

Transcription factors (TF) are also regulated by PTM that can affect transcription factor function by: 1) controlling the length of time TF are in the nucleus; 2) targeting them for degradation; 3) modulating its interaction with other proteins and/or with DNA and 4) modifying chromatin structure. A typical example of this modulation occurs in mitosis when most of the transcription factors are excluded from chromatin as a consequence of their phosphorylation, thus contributing to the attenuation of transcriptional activity in this stage of the cell cycle [124]. The re-activation of transcription during mitotic exit [125] is possibly also associated to the timely and sequential de-phosphorylation of these factors but the molecular details and the phosphatases involved are not known. Therefore phosphorylation plays a crucial role in TF regulation and some protein kinases and phosphatases have been involved in this process, although it is still an area that needs further research (Fig. 3).In this section we will review the contribution of protein phosphatases to the function of major known transcription factors.

#### 2.3.1 Myc

Myc is a transcription factor crucial for the regulation of genes related with cell proliferation, differentiation, and apoptosis. Its expression is controlled at many levels, including protein phosphorylation, which decreases DNA binding during M phase to enable proper chromatin condensation and cell cycle progression [126]. Two main phosphorylation sites are known for this protein, Ser62 and Thr58, stabilizing and destabilizing the protein, respectively. In early G1, Ser62 is phosphorylated by Ras kinases, which, in late G1, enables phosphorylation of Thr58. This status of Myc associates with the isomerase Pin1, which changes its conformation and enables PP2A binding. PP2A through its regulatory subunit B56α dephosphorylates S62, targeting Myc for ubiquitination and further degradation. Thus, PP2A acts as a tumour suppressor that negatively regulates the very well-known oncogene Myc [127,128].

#### 2.3.2 NF-κB

NF-κB (Nuclear Factor κB) family of transcription factors are involved in regulation of genes related to inflammation, immune response and apoptosis. They are usually kept inactive in the cytoplasm by inhibitory proteins (IκB) until a stimulus activates the TNF receptor, which triggers the phosphorylation of IκB kinase (IKK) which in turn will target IκB for degradation, releasing NF-κB and promoting its translocation into the nucleus to control the expression of target genes. PPM1A and PPM1B, from the PPM family of phosphatases, have been shown to dephosphorylate IKK in two specific sites (S177 and S181), thus acting as a regulator of NF-κB activity [129]. Another phosphatase, PP4, has also been found to be a regulator of NF-κB DNA binding. Although the mechanism is not fully understood, it is known that PP4 interacts with a specific member of the NF-κB family, c-Rel, and activates NF-κB-mediated transcription [130].

#### 2.3.3 Cys2His2 zinc finger proteins

Cys2His2 (C2H2) zinc finger proteins constitute the largest class of transcription factors encoded in higher eukaryotes genomes. The zinc finger modules of these proteins are usually joined by highly conserved linker sequences that are critical for DNA binding. Some studies have revealed that phosphorylation of these linker sequences by several protein kinases decreases DNA-binding affinity, interfering with gene transcription [131]. Rizkallah et al. [132] demonstrated that this phosphorylation is tightly synchronized and starts right after DNA condensation and before nuclear envelope breakdown, and it is reversed in telophase. However the nature of the phosphatases involved is not yet known.

SP1 is one zinc finger transcription factor that binds to GC-rich motifs of different promoters and regulates many cell processes including cell growth, differentiation, apoptosis, and chromatin remodelling. Its activity is also regulated by its phosphorylation status. PP2A has been identified as an important factor in the activation of SP1 by mediating the dephosphorylation of several residues, including Ser59 and Thr681 during the cell cycle, but not in mitosis [133]. SP1 dephosphorylation increases the affinity of SP1 to chromatin [134] and activates the transcription of different genes including CYP1A1 and CREM (cAMP-responsive element modulator) [135].

#### 2.3.4 p53

p53 is a tumour suppressor gene that acts as a transcription factor by regulating the expression of several cell cycle controls, DNA repair, and apoptotic genes. Several protein phosphatases are involved in the regulation of p53 levels and its role as a tumour suppressor, either directly or indirectly. One of the phosphatases widely known to control p53 stability is PTEN (Phosphatase and a tensin homologue deleted on chromosome 10). PTEN acts as an antagonist of the PI3K/AKT pathway, which allows the translocation of Mdm2 to the nucleus where it interacts with p53 and targets it for degradation. PTEN dephosphorylates and inhibits PI3K, blocking the translocation of Mdm2 to the nucleus and stabilizing p53 levels. In accordance, PTEN-depleted cells have decreased p53 levels and p53^+/−^;PTEN^+/−^ mice show similar lymphoma development as p53^−/−^ animals, highlighting an important crosstalk between the two proteins. The Wu lab also demonstrated that PTEN physically interacts with p53 and regulates its transcriptional activity by modulating its DNA binding affinity [136].

Other phosphatases have been associated to p53 regulation. Treatment with OA resulted in an increase of hyperphosphorylated p53, and treatment with a PP2A inhibitor, SV40 small t antigen, promoted p53-DNA binding and transcriptional activity [137,138]. PP2A has been shown to be responsible for dephosphorylation of Ser46 of p53, residue phosphorylated after ionizing radiation to increase p53-induced apoptotic response [139]. However, PP1 is responsible for dephosphorylating p53 at Ser15, which destabilizes p53 [140]. In addition, overexpression of PTP-S2, a nuclear tyrosine phosphatase, increased the levels and transcriptional activity of p53 [141]. Altogether these data seems to indicate that p53 levels are highly regulated by phosphorylation and that many phosphatases are involved in controlling this balance.

#### 2.3.5 Hox

The Hox gene family contribute to the morphological diversification of structures of an embryo along the head-tail axis. These genes encode for homeodomain proteins that bind to DNA and regulate the transcription of target genes. Sex combs reduced (SCR) is a *Drosophila* Hox protein that determines the formation of the labial and prothoracic segments during embryogenesis. In yeasts, a homologue of the regulatory subunit (B′/PR61) of PP2A was seen to interact and dephosphorylate the homeodomain of SCR, regulating its DNA binding affinity [142]. However, a null mutation in the PP2A-B′ gene provided no evidence in support of a role of PP2A in dephosphorylation and activation of SCR [143]. The phosphorylation status of Hox genes has been reported to influence in its DNA binding capacity [144] so it is to be expected that protein phosphatases will also be involved, although the specific ones are still not known.

#### 2.3.6 Oct4

Oct4 is a pluripotency transcription factor involved in embryonic stem cell self-renewal. Recent studies have shown that CDK1 indirectly regulates Oct4 binding to chromatin and therefore pluripotency-related gene expression. Upon mitotic entry, CDK1 activation promotes Aurora B-mediated phosphorylation of Oct4 at Ser229, resulting in Oct4 dissociation from chromatin. At mitotic exit, PP1 appears to be the responsible phosphatase to dephosphorylate Oct4 and enable its association with chromatin, increasing the transcription of targeted genes [145,146]. Liquid chromatography experiments identified 13 other phosphorylation sites for Oct4 [147], although to date only a few kinases and no specific phosphatase holoenzyme responsible for these modifications have been discovered.

## 3 Concluding remarks

The plasticity and dynamics of chromatin both during cell division and interphase is fundamental for the development of an organism and the maintenance of its health. Several of these aspects are regulated by phosphorylations. Many phosphorylation sites in several chromatin regulators have been shown to alter chromatin structure and function and a good number of kinases responsible for the modifications have been identified; however, the nature of the counteracting phosphatases is far less known and studied. Nevertheless, there is evidence showing that regulation of phosphorylation by phosphatases is as important as the responsible kinases. In the last decade we have endeavoured to increase the knowledge of the phosphatase biology by understanding the principle of their assembly, regulation and mechanism of action. We still require additional reliable methods for identifying their substrates recognition and specificity. However with the development of new technologies for the identification of phosphatase substrates combined with more precise cell engineering techniques will lead to the discovery of the major phosphatases as regulators both of chromatin dynamics and epigenetics.

## Conflict of interests

The Authors declare no conflict of interest.

## Transparency document

Transparency document. Image 1
